# A General Purpose Feature Extractor for Light Detection and Ranging Data

**DOI:** 10.3390/s101110356

**Published:** 2010-11-17

**Authors:** Yangming Li, Edwin B. Olson

**Affiliations:** Department of Computer Science Engineering, University of Michigan, 2260 Hayward St., Ann Arbor, MI 48109, USA; E-Mail: ebolson@umich.edu

**Keywords:** SLAM, LIDARs, feature detection, uncertainty estimates, descriptors

## Abstract

Feature extraction is a central step of processing Light Detection and Ranging (LIDAR) data. Existing detectors tend to exploit characteristics of specific environments: corners and lines from indoor (rectilinear) environments, and trees from outdoor environments. While these detectors work well in their intended environments, their performance in different environments can be poor. We describe a general purpose feature detector for both 2D and 3D LIDAR data that is applicable to virtually any environment. Our method adapts classic feature detection methods from the image processing literature, specifically the multi-scale Kanade-Tomasi corner detector. The resulting method is capable of identifying highly stable and repeatable features at a variety of spatial scales without knowledge of environment, and produces principled uncertainty estimates and corner descriptors at same time. We present results on both software simulation and standard datasets, including the 2D Victoria Park and Intel Research Center datasets, and the 3D MIT DARPA Urban Challenge dataset.

## 1. Introduction

The solution to the Simultaneous Localization and Mapping (SLAM) problem is commonly seen as a “holy grail” for the mobile robotics community because it would provide the means to make a robot truly autonomous. In the SLAM context, there are a variety of sensors that are commonly used, such as cameras, LIDARs, radars, infrared sensors and ultrasound sensors. Among these sensors, LIDARs are perhaps the most used, for they have the ability to accurately measure both bearing and range to objects around the robot. Additionally, they are robust to environmental noise, such as illumination and electromagnetic interference.

Generally, there are two basic approaches to mapping with LIDARs: feature extraction and scan matching. The first method extracts features (also called landmarks) from the LIDAR data; these features are added to the state vector and loops are closed using data association algorithms like Joint Compatibility Branch and Bound (JCBB) [1]. The features used often depend on the environment: in indoor settings, lines, corners and curves have been used [2–7]. Outdoors, the hand-written tree detector originally developed for the Victoria Park dataset [8] has been used almost universally (see [9–13] for representative examples). Naturally, tree detectors work poorly in offices, and corner detectors work poorly in forests. The lack of a general-purpose feature detector that works well in varied environments has been an impediment to robust feature-based systems.

The alternative LIDAR approach, scan matching, directly matches point clouds. This approach dispenses entirely with features and leads to map constraints that directly relate two poses. Scan matching systems are much more adaptable: their performance does not depend on the world containing straight lines, corners, *or* trees. However scan matching has a major disadvantage: it tends to create dense pose graphs that significantly increase the computational cost of computing a posterior map. For example, suppose that a particular object is visible from a large number of poses. In a scan matching approach, this will lead to constraints between each pair of poses: the graph becomes fully connected and has *O*(*N*^2^) edges. In contrast, a feature based approach would have an edge from each pose to the landmark: just *O*(*N*) edges.

Conceptually, the pose graph resulting from a scan matcher looks like a feature-based graph in which all the features have been marginalized out. This marginalization creates many edges which slows modern SLAM algorithms. In the case of sparse Cholesky factorization, Dellaert showed that the optimal variable reordering is *not* necessarily the one in which features are marginalized out first [14]: the information matrix can often be factored faster when there are landmarks. Similarly, the family of stochastic gradient descent (SGD) algorithms [15,16] and Gauss-Seidel relaxation [17,18] have runtimes that are directly related to the number of edges. The extra edges also frustrate sparse information-form filters, such as SEIFs [19] and ESEIFs [20,21].

Feature-based methods have an additional advantage: searching over data associations is computationally less expensive than searching over the space of rigid-body transformations: as the prior uncertainty increases, the computational cost of scan matching grows. While scan matching algorithms with large search windows (*i.e.*, those that are robust to initialization error) can be implemented efficiently [22], the computational complexity of feature-based matching is nearly independent of initialization error. And finally, feature-based methods tend to work better outdoors, because they often reject ground strikes that result when the robot pitches or rolls.

In short, feature-based methods would likely be preferable to scan matching if they were able to offer the same robustness and broad applicability to different environments. Because of the advantages of feature-based SLAM solutions, the extraction of features from LIDAR data has been extensively explored.

As pointed out before, classical feature detectors rely on prior knowledge of environments. Researchers reconstruct an environment with lines and curves based on previously collected environmental information and assumptions, then features are extracted from the lines and curves. For example, a specific line fitting algorithms [23,24] will be carefully tuned to the characteristics of an environment (mainly on the contour size and the error threshold). After lines are re-constructed, the features with stable positions, for example, midpoints of lines or intersection points of lines, will be extracted as features. These classical feature detectors are easy to implement and have excellent performance in correspondingly target environments, but they are not widely applicable to other types of environment.

Recent work on feature detectors focuses on addressing these problems. The curvature estimation based feature extractor [2] tries to fit various environments with curves [2]. The B-spline based extractor [25] represents the world with B-splines; although it is generally applicable, the segmentation of laser data, the selection of control points, and the feature representation in the data association process are still areas of active research [25].

Zlot and Bosse [26] propose a number of heuristics for identifying stable keypoints in LIDAR data. Their methods begin with clustering connected components and then either (1) computing the centroids of each segment, (2) computing the curvature of each segment, or (3) iteratively computing a locally-weighted mean position until it converges. Our approach replaces these three mechanisms with a single method. Zlot and Bosse additionally investigate descriptor algorithms, which significantly simplify data association tasks. These descriptor methods could also be applied to our detector.

**Figure 1.**
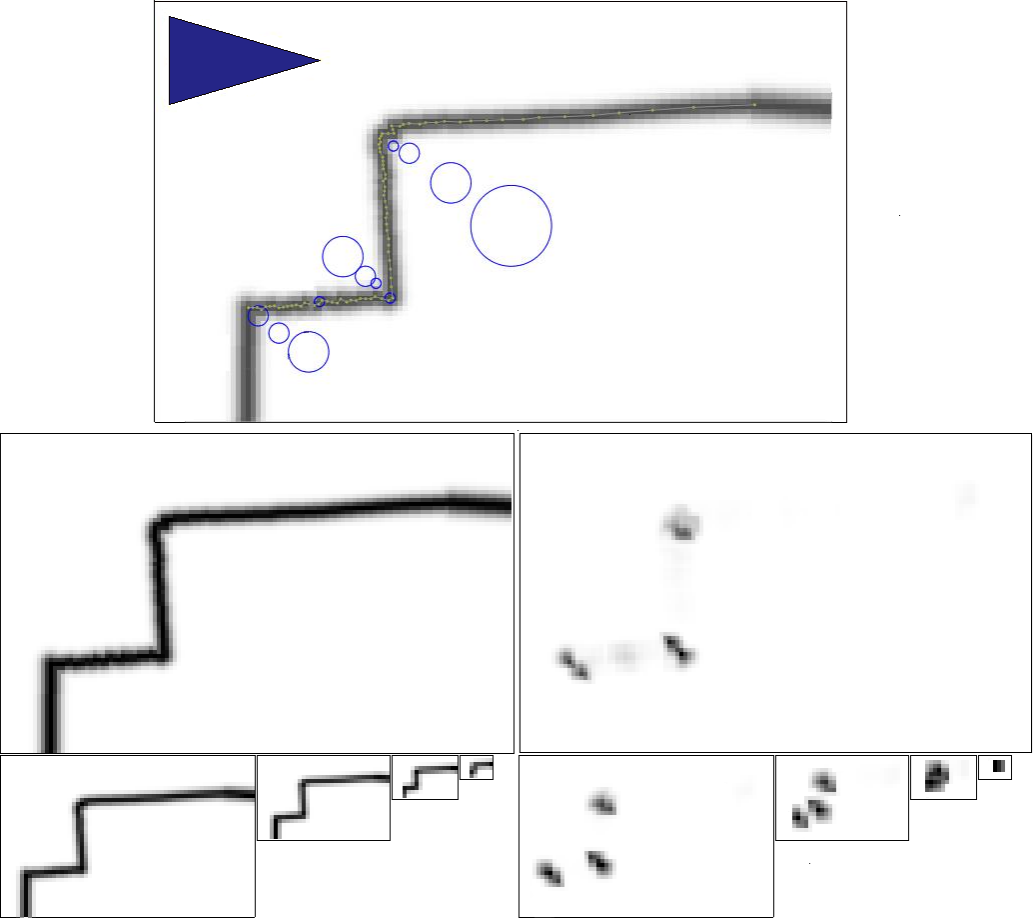
Multi-scale feature extraction from LIDAR data. Our method rasterizes LIDAR data and applies the Kanade-Tomasi corner detector to identify stable and repeatable features. Top: the input image with overlaid local maxima (prior to additional filtering). Circles indicate features, with the radius equal to scale of the feature. Left: image pyramid of input. Right: Corner response pyramid, where local maxima indicate a feature.

In this paper, we describe a general purpose feature detection algorithm that generates highly repeatable and stable features in virtually any environment, as shown in Figure 1. Our approach builds upon methods used in image processing, where the need for robust feature detectors has driven the development of a wide variety of approaches. In particular, we show how the Kanade-Tomasi [27], a variant of the Harris corner detector [28] can be applied to LIDAR data. At the same time, we also studied the characteristics of the features extracted using our method, including uncertainties and feature descriptors.

The central contributions of this paper are:

- We propose a general-purpose feature detector for 2D and 3D LIDAR data by adapting the Kanade-Tomasi corner detector.
- We show how to estimate feature uncertainties as well as feature descriptors.
- We show how to avoid false features due to missing data, occlusion, and sensor noise.
- We present experimental evidence that our methods work consistently in varied environments, while two traditional approaches do not.

In the next section, we describe how we convert 2D and 3D LIDAR data into images for feature detection. In Section III, we describe how to extract features from pretreated LIDAR data. In Section IV, we describe how uncertainty information and feature descriptors can be obtained. In Section V, we present experimental evaluations of our methods versus standard methods.

## 2. Rasterization of LIDAR Data

Our method is inspired by the success of feature detectors in the image processing methods field. The core idea is to convert LIDAR data into an image that can then be processed by proven image processing methods. Obviously, this process must take into account the fundamental differences between cameras and LIDARs.

### 2.1. Challenges in Proposed Method

A camera image samples the intensity of a scene at roughly uniform angular intervals. Individual pixels have no notion of range (and therefore of the shape of the surface they represent), but the intensity of the pixels is assumed to be approximately invariant to viewpoint and/or range. As a consequence, the appearance of a feature is reasonably well described by a set of pixel values.

LIDARs also sample the scene at uniform angular intervals, but each sample corresponds to a range measurement. Critically, unlike cameras, the value of each “range pixel” is profoundly affected by the position and orientation of the sensor. As a result, it becomes non-trivial to determine whether two features encoded as a set of <angle, range> tuples match.

**Figure 2.**
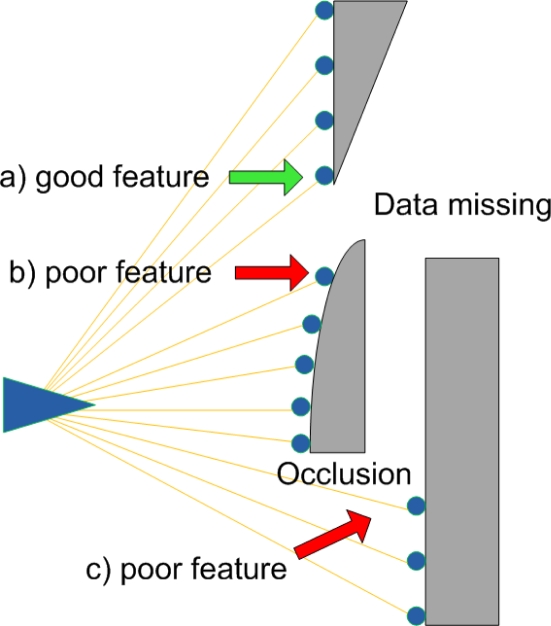
Feature detection scenarios. The direction from which a surface is viewed is critical to identifying sharp features. In case (a), a sharp corner must exist; in contrast, case (b) may not be a well-defined feature. Our method addresses this issue by rendering the worst-case (most featureless) shape, rather than attempting to threshold the angle of the hidden corner. In case (c), a foreground object’s shadow can cause a false boundary to appear on a background object; this case is handled explicitly.

Because of these fundamental differences between cameras and LIDARs, there are some challenges if we want to extract features from LIDAR data using extractors from the computer vision field. Figure 2 illustrates these challenges. The greater noise of LIDARs compared to cameras presents the first challenge. We model the noise as:

(1) $$\begin{array}{c} Q\approx 𝒩(\mu_{Q},\sum_{Q})\\ \mu_{Q}=\left[\begin{array}{l} 0\\ 0 \end{array}\right],\;\;\sum_{Q}=\left[\begin{array}{cc} \sigma_{r}^{2} & \sigma_{r\alpha }\\ \sigma_{r\alpha } & \sigma_{\alpha }^{2} \end{array}\right] \end{array}$$

where *r* and *α* denote observation range and angle, respectively.

Normally, the range noise is at centimeter magnitude, for example, SICK-LMS 291-S05 has range resolution of 10 mm, and a statistical error standard deviation of 10 mm under typical conditions; and the angular noise is negligible [10]. Although LIDAR is much more precise than ultra sound and radar sensors, the noise is still large enough to induce false positives. Another source of noise is discretization error. The angular resolution of LIDAR is within the range of 0.25° to 1.00° [10]. Due to the fact that information between two adjacent observation points are missing, the positioning error would be introduced into the feature extractor. Because the proposed method is trying to capture distinct physical characteristics of observable surfaces, elimination of feature candidates that derive from noise is highly desirable.

LIDAR data also suffers from the missing data problem. The problem results when some parts of obstacles are not visible due to occlusions. These occlusions can also create false features, such as where a foreground occluder makes it appear that an abrupt shape change occurs on a background object.

**Figure 3.**
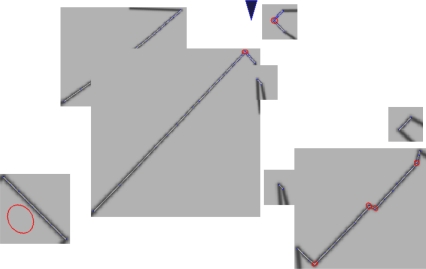
Intel Research Center rasterization. This indoor dataset has many rectilinear features. Rendering is performed for each contour individually as illustrated by the black rectangular outlines; this dramatically reduces computation time. Ellipses indicate 3σ uncertainty bounds for detected features. At the occlusion boundaries of each contour, the conservatively extrapolated contour is readily visible.

To conquer these challenges, we have chosen to rasterize LIDAR data by projecting the LIDAR points into a 2D Euclidean space. The resulting image roughly corresponds to viewing the scene from above (see Figure 3). This choice of representation restores the invariance properties upon which computer vision methods rely, though this choice also creates new challenges. This section will describe how we approach these challenges.

### 2.2. 2D Data

#### 2.2.1. 2D Data Rasterization

2D data is rendered into images using a Gaussian kernel smoothing filter, as shown in Figure 3. The Gaussian kernel has several practical advantages. At short ranges, the range noise of the sensor can cause smooth surfaces to appear rough. This, in turn, causes false feature detections. The Gaussian kernel essentially smoothes these surfaces, preventing features from being detected.

Specifically, points that are close together appear to be part of a much rougher (and thus feature-rich) surface, while points that are far apart appear to be part of a very smooth surface. Our rasterization process addresses this problem by computing the uncertainty and rendering laser features with a corresponding Gaussian. Apparently, the width of the Gaussian kernel is a function of both the LIDAR’s observation noise, Σ*_Q_*, and the positional uncertainty that arises from sparsely sampling a surface. In other words, even if a sequence of three LIDAR points that are not contaminated by observation noise implies the existence of a corner, the actual position of the corner is uncertain due to the spacing between the points. Compared with discretization error, the angular observation noise is trivial. Therefore the spatial resolution of a measurement at range *r* is just *r* sin(Δ*θ*), where Δ*θ* is the angular resolution of the sensor (typically 1 degree for a SICK sensor). The width of the Gaussian kernel (which changes for every measurement) reflects the sum of these uncertainties:

(2) $$\sigma^{2}\approx \sigma_{s}^{2}+{\left(r\;\text{sin}(\Delta \theta )\right)}^{2}$$

**Figure 4.**
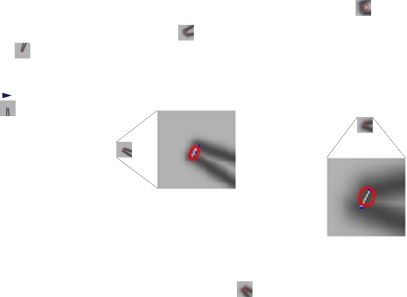
Victoria Park Rasterization. This figure shows rasterization for a 2D LIDAR scan from an outdoor, tree-filled environment. The same rendering parameters were used for both the Intel and Victoria Park datasets.

We also addressed the other two challenges with the proper rasterization method:

- Missing data: often, the full shape of a contour is not visible, which can lead to feature detections at the visibility boundary. In some cases, the *absence* of LIDAR data is proof that a strong feature is present (Figure 2(a)), while in other cases, it is possible that there isn’t a strong feature at all (Figure 2(b)). While we could attempt to explicitly measure and threshold the angle of the hidden corner, this process would add a number of hard-to-tune parameters. (Estimating the angle of the observed contour, for example, is sensitive to noise in the individual range measurements). Our approach is to render the most conservative (*i.e.*, the smoothest) contour consistent with the observed data. This conservative contour is then passed to our system without additional modification. For each contour, we draw two comet tails that start from the two boundary points and are along the observation direction (Comet tails are indicated as blurred black lines in Figures 3 and 4). These two comet tails indicate how sharply the boundary angles are. Therefore, sharp boundaries are extracted as feature candidates.
- Occlusion: A foreground object can occlude portions of a background object (see Figure 2(c)) making it appear as though the background object has an abrupt boundary. Our approach is simply to suppress feature detections that are close to these occlusion boundaries.

Rasterization inevitably introduces additional quantization noise. However, this quantization noise is modest in comparison to the range noise of sensors, and is small in comparison to the uncertainty arising from sampling effects. In our experiments, for example, we used an image resolution of 2 cm per pixel.

In short, we render the most conservative contours from raw LIDAR data, connect adjacent points that ostensibly belong to the same physical object with straight lines, render lines with a Gaussian kernel low pass filter, draw comet tails for each contour and reject false positives induced by occlusion. The rasterization results are shown in Figures 3 and 4.

#### 2.2.2. Rasterization Optimization

In the case of many 2D datasets, the bulk of the rendered image is empty. It is possible to significantly accelerate the feature detection step by rendering each contour into separate, smaller images. On datasets like Victoria Park, in which there are large amounts of empty space, this technique provided an average speed up of 31.8 times.

Rendering each contour separately also makes it much easier to suppress errant feature detections caused by the conservative surface extrapolations described above; since each contour is rendered separately, there is no possibility that the extrapolated surface will intersect another contour, creating a feature.

### 2.3. 3D Data

The proposed method is also applicable to 3D LIDAR data. While this conclusion might be counter-intuitive, we found 3D data is easier to process than 2D data. This is because 3D LIDARs acquire far more information from environments than 2D LIDARs. For example, 3D sensors often see over obstacles, which reduces the severity of the occlusion problem. However, we can not render images based on where we obtained LIDAR returns, as what we do to 2D data, because 3D laser sensors obtain samples almost everywhere. We rasterize 3D data using following method:

1. Divide the horizontal observable plane, *H* ⊂ ℝ^2^, into square grids, *g*_0_, . . .,*g_n_*,. . ., let(*x_n_*, *y_n_*) denote the left-down corner of *nth* grid and *δ* denotes the length of *x* and *y* edge;
2. Orthogonally projects all laser observation points, *p*_0_, . . ., *p_m_*,. . ., into grids, and forms point sets *B*_0_, . . ., *B_n_* . . ., *B_n_* = {*p_m_*|*x_n_* ≤ *x_m_* ≤ *x_n_* + *δ*, *y_n_* ≤ *y_m_* ≤ *y_n_* + *δ*}, where *x_m_*, *y_m_* are *x*, *y* coordinates of point *p_m_*;
3. Find the two points that have largest and smallest z coordinate values for every point set, *B_i_*, and convert every grid to the corresponding pixel, in which the gray shade of the pixel is proportional to the difference between the z coordinates of the two points.

In other words, we render each pixel according to the range of heights collected for that pixel. If three LIDAR samples are collected in the area corresponding to a single (x,y) pixel with z = 1, 1.5, and 2.5, the maximum difference in height is 1.5. We render 3D data with this method because the procedure effectively measures the visible height of the objects around it and is invariant to viewpoint, thus the method has an explicit physical meaning. However, this procedure requires a fair number of LIDAR returns for each pixel, which necessitates a coarser spatial resolution.

**Figure 5.**
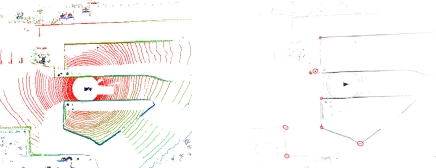
3D Scan Rasterization. Left: a Velodyne scan with points colored according to Z height. Right: Rasterized image with superimposed extracted features and corresponding uncertainties. 3D LIDAR data was rasterized by considering the range of Z values in each cell of a polar grid.

Note that we assume that the robot can measure its pitch and roll with respect to the gravity vector with reasonable accuracy; the cost of such a sensor is inconsequential in comparison to that of any laser scanner. When projecting points, the pose of the vehicle is taken into account; as a consequence, the resulting images are invariant to roll and pitch. The rasterization effect of Velodyne data came from MIT DARPA Grand Challenge [29] can be seen in Figure 5.

## 3. Feature Detection

### 3.1. Feature Detector Selection

After rasterizing LIDAR data into images, the next task is to identify stable and repeatable features. Generally, our images are much simpler than ordinary images, because our images only contain intensities. Therefore, we consider low-level corner detection algorithms, because they make few assumptions about the underlying data. From low-level corner detection algorithms, the Kanade-Tomasi corner detector [27] was selected for following reasons.

- It is highly consistent [30] making extracted features are highly repeatable;
- It has strong discrimination between corners and edges [27], which naturally matches the goal of the proposed method;
- It is rotationally invariant when the image convolve with a circular filter, because our images are constructed using Gaussian kernels, the proposed method naturally meets this condition;
- It has relatively low computation complexity;

### 3.2. Kanade-Tomasi Corner Detector

The Kanade-Tomasi corner detector defines corners as pixel patches whose self-similarity is sharply peaked. The KT detector simply checks the weighted sum of the square difference between an image patch *I*(*u*, *v*) and the counterpart patch shifted by (*x*, *y*). After the approximation of the shifted patch with Taylor expansion, the problem is simplified as:

(3) $$\begin{array}{c} S(x,y)\approx \left(\begin{array}{cc} x & y \end{array}\right)A\left(\begin{array}{l} x\\ y \end{array}\right)\\ A=\sum_{u}\sum_{v}w(u,v)\left[\begin{array}{cc} I_{x}^{2} & I_{x}I_{y}\\ I_{x}I_{y} & I_{y}^{2} \end{array}\right]=\left[\begin{array}{cc} \langle I_{x}^{2}\rangle & \langle I_{x}I_{y}\rangle \\ \langle I_{x}I_{y}\rangle & \langle I_{y}^{2}\rangle \end{array}\right] \end{array}$$

and all pixels that have two strong eigenvalues are extracted as corners.

The Kanade-Tomasi corner detector is virtually identical to the Harris corner detector [28], with the exception that the minimum eigenvalue of the structure tensor is computed exactly, rather than approximated. We achieved noticeably better results from the Kanade-Tomasi detector.

### 3.3. Multi-Scale Feature Detection

Our system detects features at a variety of scales so that we can exploit features that are both physically small and large. To do this, firstly, we build a power-of-two image pyramid for the original rasterized image. Secondly, we process each of these images with KT corner detector. In the processing procedure, KT detector builds structure tensors that are the sum of squared differences of image patches, and uses the minimum eigenvalue as the indicator to the strength of a corner. Thirdly, the local maxima are selected as corners. We perform KT corner detection on each level of a power-of-two image pyramid, extracting corners wherever local maxima occur.

This feature-detection scheme is very similar to that used by the SURF detector [31]. Our implementation down-samples images using a σ = 1.0 Gaussian kernel of width 5. Corners are additionally subjected to eigenvalue threshold of 0.2. This design parameter is fairly robust: the system works well on a variety of datasets over a range of values.

While we want to detect features at multiple scales, we do not want to match these features in a scale invariant manner: unlike cameras, LIDARs directly observe the scale of the objects in the environment. Thus, unlike camera-based methods, the scale at which we detect an object is useful in data association.

## 4. Feature Description

### 4.1. Feature Uncertainty Evaluation

The positional uncertainty of features is of critical importance to SLAM applications. It has been shown that the covariance matrix of a Kanade-Tomasi Corner is the inverse of the structure tensor [32]. Our use of variable-sized Gaussian kernels when rasterizing the LIDAR data encodes the spatial uncertainty in the image, and this is reflected in the structure tensor. All that remains to be done is to scale the covariance matrix according to the square of the resolution of the image (in meters per pixel).

The covariance estimates produced by our system can be seen in Figures 3 and 4 for 2D data, and in Figure 5 for 3D data. The ellipses correspond to 3σ confidence intervals. The fact that principled covariance estimates can be easily derived is one of the strengths of our method.

### 4.2. Feature Descriptor

Feature descriptors attempt to concisely describe the environment in the region around a feature detector. These descriptors aid data association. In the computer vision literature, descriptors are widely used, SIFT [33] and SURF [31] are well-known examples.

In order to be invariant to viewpoint, descriptors for images must be invariant to rotation, scale, and affine distortion. The requirements for a LIDAR descriptor, however, are quite different. LIDARs directly measure ranges, and thus there are no affine distortions, nor is there scale ambiguity. (It is still useful to detect features on multiple scales, however we do not require our descriptors to be invariant to scale.) In fact, of the invariance usually described for images, we only desire rotational invariance for LIDAR data. In this paper, we consider two descriptors.

#### 4.2.1. SIFT-Style Descriptor

The SIFT descriptor is a proven method that computes a 128-element histogram of the gradient in a pixel patch around detected features. However, a simpler descriptor is desired in our application, because of both the simplicity of the laser image and the desire for low computational complexity. Experimentally, a 2 × 2 × 8 = 32 descriptor is good enough to capture the characteristics of corners in our application. Except for the difference on the size of a descriptor, the method we adopted in the paper is identical to the recommended SIFT descriptor [33].

#### 4.2.2. Simple Angle Descriptor

Unlike cameras, LIDARs capture the physical shape of an obstacle. Therefore, the rasterized images from laser data mainly contain curves. Noticing this characteristic, we propose a much simpler descriptor, which utilize angles around a corner, to describe corners.

**Figure 6.**
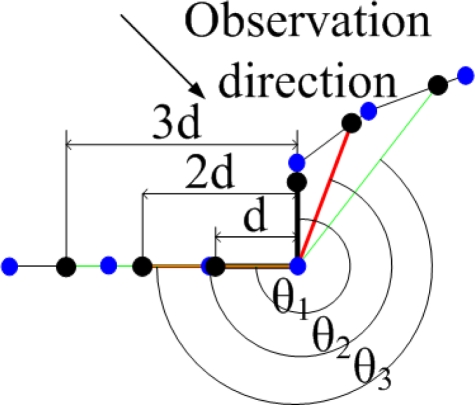
Feature descriptor. Blue points denote raw laser observations and black points indicate the points that are *d*, 2*d* and 3*d* meters away from the extracted corner.

The simple descriptor consists of four elements, each of them an angle. To generate this descriptor, we use a parameter *d*, which indicates the fineness with which we describe the extracted corner. Secondly, we find three sets of points (each set contains two points, shown as the black points in Figure 6 that lie on the rendered lines from raw laser data, and at the same time, the distances between the three point sets and the extracted corners are *d*, 2*d* and 3*d*, respectively. Thirdly, every two points in a set from an angle, along with the extracted corner, and the three angles corresponding to *d*, 2*d* and 3*d* are the first three elements in the simple descriptor, denoted as *θ*_1_, *θ*_3_, *θ*_3_ in Figure 6. The fourth element is the heading of the corner. In mapping applications, a robot roughly knows the pose of itself, thus the heading of a corner is also roughly known. This information is very useful in an environment with high self-similarity. Particularly, the fourth element equal to the heading of the internal bisector, which belongs to the angle formed by the average of the three angles.

To sum up, the simple angle descriptor contains four angles, the first three are the angles formed by the extracted corner and the three point sets that are *d*, 2*d* and 3*d* meters away from the extracted corner, and the fourth angle is the heading of the corner. Notice that the fourth element in the descriptor makes the descriptor highly descriptive in robot mapping applications, but, at the same time, it also makes the descriptor orientation-variant. In other applications where the corner heading is not known in advance, we can simply set the fourth element to zero to retain the orientation-invariance. Figure 6 gives a graphical explanation of the simple angle descriptor. In the figure, blue points denote original laser observations and black points indicate points used for generating a descriptor.

The only parameter that have impact on the descriptor is the distance, *d*. Generally, larger *d* values will have comparatively stable angle and heading values, but requires bigger areas to form a descriptor; smaller *d* values will suffer more significantly from noise. In our experimental environments, the ideal value of *d* is between 0.05 meter and 0.10 meter.

## 5. Results

While there is no universally agreed-upon definition of a corner, a good corner detector should always detect highly repeatable and stable features. This is especially true in a SLAM context, since repeatability (the consistency with which a given landmark is observed) is critical. Each re-observation of a landmark creates a “loop closure”, improving the quality of the posterior map.

### 5.1. Software Simulation

The proposed method was first verified by software simulation, which allows us to measure the performance of the proposed method when both the true positions of corners and the robot are known. In real environments, these data are inevitably contaminated by noise.

**Figure 7.**
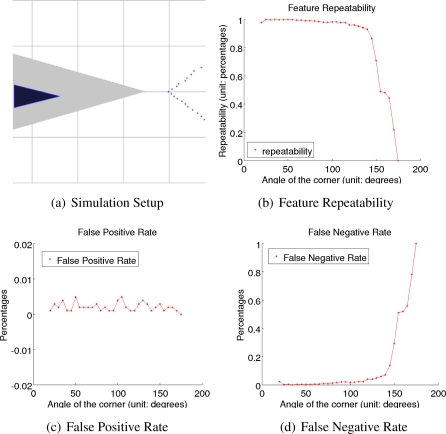
Feature repeatability and detection rates. In **(a)**, the black triangle denotes the simulated robot position, which was randomly localized in the gray area; blue points are simulated laser scans. In **(b)**, the red curve indicates the repeatability in the simulation. In **(c)** and **(d)**, curvatures indicate the false positive detection rate and the false negative detection rate, respectively.

We set up a simple environment that contains only one physical corner, as shown in Figure 7(a), therefore avoiding errors due to data association processes. In the figure, the robot indicated by the blue triangle is randomly located in the possible area, which is indicated by the gray triangular area in Figure 7(a). The simulated obstacle, as the red lines show, is always observable to the robot when the robot heading is within the range from −10*°* to 10*°*. Blue points indicate simulated observation, which are generated according to the noise model shown in Equation (1). Specifically, the standard deviation of range error and angle error are 10 mm and 0.1*°*, and the angular resolution is 1°.

The detection rates of the proposed method, including the overall detection rate, the false positive rate and the false negative rate, were studied in the simulated environment. The overall detection rate that equals to the number of re-observations over the number of simulation steps are shown in Figure 7(b); the false positive rate that equals to the number of false features over the number of simulation steps are shown in Figure 7(c), and the false negative rate that equals to the number of missed features over the number of simulation steps are shown in Figure 7(d). The figures show not only that the proposed method has high detection rates, but also that the false detection rate is low, mainly because of rasterizing data with reasonable Gaussian low pass filters.

**Figure 8.**
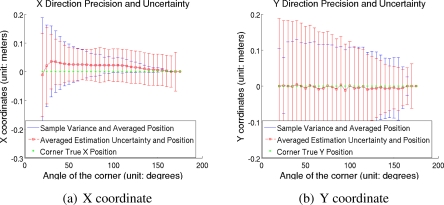
Feature precision. The x direction and the y direction precisions were demonstrated in (a) and (b), respectively. Blue solid lines, red dash lines and green star marks indicate sample variances, estimated variances and true corner positions, respectively.

Next, we studied the precision of the proposed feature detector. Figure 8(a), 8(b) show the x direction and the y direction precision of the detected features, respectively, along with the averaged uncertainty estimations and sample variances. The figure shows that the detected features have high precision in relation to the observation error of a LIDAR, and that the estimated uncertainty are close to sample variances.

### 5.2. Experiment on Classical Dataset

The proposed method was also applied to classical laser datasets, including the Intel Research Center dataset (indoor 2D), the Victoria Park dataset (outdoor 2D) and the MIT DARPA dataset (outdoor 3D).

Firstly, we measure the repeatability of the proposed method using the two 2D datasets. For these datasets, we used posterior position estimates produced by conventional SLAM methods; this “ground truth” allowed us to test whether a particular landmark should have been observed given the location of the robot. When evaluating whether a landmark was correctly observed, we used a simple nearest-neighbor gating rule: if a feature was observed within a distance *d*_1_ of an existing landmark, the two were associated. If the feature was more than *d*_2_ away from the nearest landmark, a new landmark was created. Features between *d*_1_ and *d*_2_ were discarded. On the Intel data set, we used *d*_1_ = 0.1 m, and *d*_2_ = 0.3 m, and on the Victoria Park dataset, we used *d*_1_ = 1.0 m, *d*_2_ = 3.0 m.

In addition to our proposed method, we provide two comparison methods (both of which used the same data association procedure):

- Corner detector: Line segments are extracted from nearby points using an agglomerative method. If the endpoints of two lines lie within 1.2 m of each other, and the angle between the two lines is between 75 and 105 degrees, a corner is reported. This particular method is adapted from [34].
- Tree detector: The standard method of tracking features in Victoria park is using a hand-written tree detector with hand-tuned parameters [8]. We used this detector with no additional modifications.

**Figure 9.**
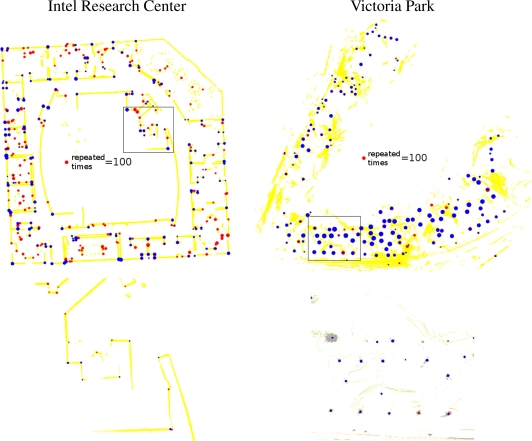
Repeatability. Top: the locations of detected features are shown; the size of each marker represents the number of re-observations (large dots denote often-reobserved features). Bottom: larger views of the framed parts of the top figures. The proposed method matches or exceeds the performance of the corner and tree detectors even in the environments for which those detectors were designed. While the performance of the corner and tree detectors is very poor in the “wrong” environment, the proposed method is robust in both environments.

**Figure 10.**
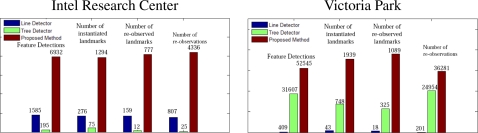
Feature detection performance. Three detectors ran on two different datasets: Intel Research Center (indoor) and Victoria Park (outdoor). New features were instantiated when a new detection was far away from any previous landmarks. A larger number of loop closures (re-observations) generally leads to better maps. Four important indicators for feature detectors were compared, which are feature detection amount, instantiated landmark amount, re-observed landmark amount and the re-observation amount. The two histograms show that our method outperforms the other two methods even in the environments for which the specialized methods were designed.

As shown in Figure 9, the performance of the proposed method is generally as good or better than the other detectors. In contrast, while the performance of the tree detector is good in the Victoria Park dataset, it is very poor indoors (in the “wrong” environment). Similarly, the performance of the corner detector is good in the Intel dataset and poor in Victoria Park. Our general-purpose method performs well in both environments, as indicated in Figure 10.

**Figure 11.**
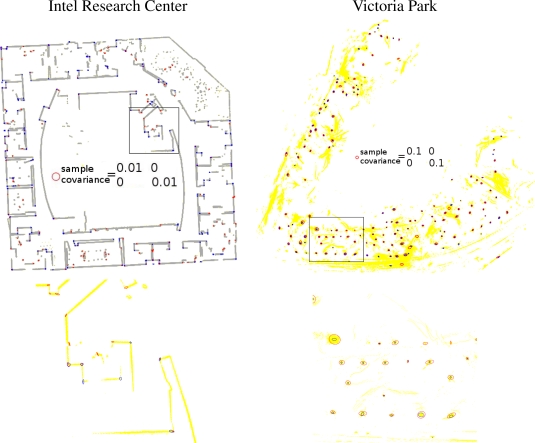
Uncertainty. Top: the sample variance of detected features is shown; the size of each ellipse corresponds to 3σ confidence. Bottom: Larger views of the framed parts of the top figures.

Next, we study the precision of the proposed feature detector. We use sample covariances as the indicator to the precision of our feature detector, for we do not know the true positions of features in the datasets. As the comparison, we also shows the averaged covariance estimations to verify our estimation to feature covariances. Figure 11 shows the comparison. The ellipses correspond to 3σ uncertainties. Notice that for the proposed method, there are fewer ellipses than points in Figure 9, because the variances of some landmarks are too small to be drawn.

**Figure 12.**
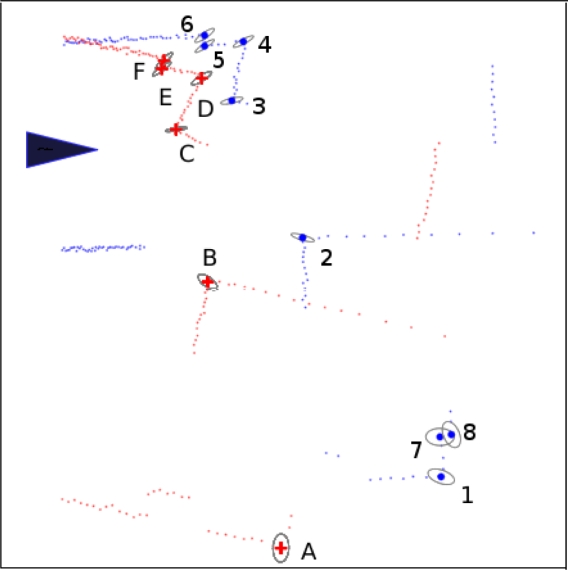
Detected Features at Two Different Time Points. Red plus mark and blue red points indicate two sets of features that were detected at different time points through the proposed method. The observation positions are indicated by the black triangle. Grey ellipses indicate 3*δ* uncertainties and numbers and letters are index of features. The ground truth data is that A=1, B=2, C=3, *etc*. Feature 7 and 8 has no corresponding feature, due to having a different view of the environment.

Next, we verified our feature descriptions, including uncertainty estimation and descriptors. In Figure 12, we show the extracted features, denoted by colored solid points, along with the estimated uncertainty, denoted by ellipses. From the figure we can easily see that the physical corners are located in the 3σ confidence ellipses, which means that uncertainty evaluation is reasonable. Tables 1, 2 show the feature similarities indicated by the SIFT-style descriptors and the simple angle descriptors between two sets of observations, which are indexed in Figure 12. The table clearly shows same features have very similar descriptors while descriptors of different features have great differences, even when they are much more computationally efficient than the SIFT descriptor.

Lastly, we applied the proposed method to the MIT DARPA dataset. Due to the fact that the MIT DARPA dataset does not have many loops, the repeatability, sample variance and false positive rate can not be computed accurately. Therefore, we only show the extracted features with the 3*δ* uncertainties in Figure 5. Still, we can see the effectiveness of the proposed method from the reasonable distribution of features and the size of uncertainty ellipses.

## 6. Conclusions

SLAM methods that use features have potential advantages over pose-to-pose methods due to the greater sparsity resulting from having landmarks. However, while feature-based methods are virtually the only viable approach in vision applications, feature detectors for LIDAR data have generally been designed for specific environments and are thus of limited general applicability.

We have described rasterization methods for 2D and 3D laser scans that allow computer vision methods to be applied to LIDAR data. We demonstrate the general-purpose applicability of the proposed method on benchmark indoor and outdoor datasets, where it matches or exceeds the performance of specialized detectors. The proposed method is also capable of producing uncertainty estimates and feature descriptors, which are two critical factors for SLAM applications. With software simulation and experiments in classical datasets, we verified the effectiveness and efficiency of the proposed method. The experimental results support that the proposed method outperforms the two special designed feature detectors—the line corner detector and the tree detector in both classical indoor and classical outdoor environments.

In future work, we plan to more deeply explore the rasterization issues arising from 3D data and to study the repeatability of the proposed method in varied 3D data (for example, 3D data collected with a nodding LIDAR).

## Figures and Tables

**Table 1.** SIFT-style Descriptor Similarity. This table lists Euclidean distances in radians, between the SIFT-like descriptors of detected features, shown in Figure 12. In each case, the correct match has very small descriptor distance.

	1	2	3	4	5	6	7	8
A	**0.87**	6.33	1.31	11.73	16.21	19.56	18.33	19.21
B	4.31	**0.56**	6.17	13.72	17.42	16.09	14.66	17.31
C	2.65	1.34	**1.53**	14.03	15.36	16.80	21.01	19.74
D	9.58	15.47	13.58	**1.04**	18.90	19.01	16.92	19.03
E	14.89	18.01	14.12	16.61	**2.07**	2.41	1.99	3.36
F	18.55	15.46	16.90	18.08	1.92	**1.81**	2.67	3.06

**Table 2.** Simple Angle Descriptor Similarity. This table lists Euclidean distances in radians, between the proposed angle descriptors of detected features, shown in Figure 12. In each case, the correct match has very small descriptor distance.

	1	2	3	4	5	6	7	8
A	**0.32**	5.75	6.89	2.22	5.85	0.94	7.25	0.95
B	6.29	**0.26**	1.10	8.17	0.76	5.46	1.45	6.72
C	6.99	1.20	**0.32**	8.92	1.15	6.18	0.58	7.11
D	1.61	7.37	8.55	**0.75**	7.46	2.28	8.92	1.03
E	6.21	0.57	1.14	8.10	**0.07**	5.38	1.50	5.01
F	1.46	5.59	6.72	2.42	5.69	**0.41**	7.08	0.96
